# Association between anosognosia and neuropsychiatric symptoms in Alzheimer’s disease dementia patients

**DOI:** 10.1038/s41598-025-29569-z

**Published:** 2025-12-04

**Authors:** Michael Elnemais Fawzy, Sharon Wang, Phebe Palmer, Jennifer Gatchel, Gad A. Marshall, Geoffroy Gagliardi, Patrizia Vannini

**Affiliations:** 1 Massachusetts General Hospital, Boston, MA USA; 2 Brigham and Women’s Hospital, Boston, MA USA; 3 Harvard Medical School, Boston, MA USA

**Keywords:** Awareness, Alzheimer’s disease, Anosognosia, Neuropsychiatric symptoms, Neuroscience, Psychology, Neurology

## Abstract

Anosognosia, the lack of awareness of memory decline, and Neuropsychiatric Symptoms (NPS) are prevalent and debilitating symptoms in Alzheimer’s disease (AD) dementia. Understanding the coexistence of these symptoms may help guide clinical interventions and treatment strategies. This study aimed to compare NPS prevalence in patients with and without anosognosia at baseline and to assess the association between anosognosia and NPS over time. We examined patients with AD dementia enrolled in the Alzheimer’s Disease Neuroimaging Initiative (ADNI). To be included in the current study, patients had to have undergone baseline assessments and at least one subsequent follow-up evaluation. Furthermore, all patients had to have amyloid (as assessed using Positron Emission Tomography, PET), Mini-Mental State Examination (MMSE), Neuropsychiatric Inventory (NPI), and Everyday Cognition (ECog) variables available throughout the study. Anosognosia, our exposure of interest, was determined using Ecog scores from patients and study partners. Study partners evaluated the presence or absence of 12 NPS (our outcomes of interest) using the NPI. Cox proportional hazards models, excluding patients who had any symptoms of NPS at baseline, were used to evaluate NPS onset by group (anosognosia/no anosognosia) while adjusting for age, sex, years of education, and MMSE. 112 patients with follow-up data (mean = 1 year) were included in this study. Of these, 47.3% (*n* = 53) had anosognosia, while 52.7% (*n* = 59) did not. In those with anosognosia at baseline, we observed a trend toward greater prevalence of agitation and motor symptoms. Exploratory time-to-event analysis demonstrated that the patients with anosognosia had a faster onset of apathy (HR: 2.78, 95% CI: 1.37–5.62, *p* = 0.01) compared to the patients without anosognosia. In this exploratory study, while there were no significant differences in frequency of NPS at baseline between the groups, patients with anosognosia demonstrated faster onset of apathy as compared to patients without anosognosia. These findings underscore the importance of longitudinal assessments and tailored interventions targeting the management of NPS in AD dementia patients with anosognosia. Further research is warranted to explain the underlying mechanisms driving these associations and to inform the development of targeted therapeutic strategies aimed at improving patient outcomes in this population.

**Supplementary Information:**

The online version contains supplementary material available at 10.1038/s41598-025-29569-z.

## Introduction

Alzheimer’s disease (AD) is a progressive neurological ailment characterized by cognitive deterioration, particularly in memory, that significantly impacts both patients and their caregivers^1^. While some patients are aware of their cognitive decline, previous research has shown that up to 80% of patients diagnosed with AD dementia experience a lack of awareness of their cognitive decline^2,3^, a condition known as anosognosia^4^. Moreover, some studies have shown that awareness decreases as the disease progresses^5–14^ but see e.g^15^. Several studies have demonstrated that anosognosia in AD is associated with increased caregiver burden^16^, and poorer treatment adherence, and may contribute to underdiagnosis or delayed diagnosis of AD, potentially limiting access to early interventions and treatments^17^.

Additionally, other frequently observed symptoms in AD include neuropsychiatric symptoms (NPS), and the severity of these symptoms also tends to increase as the disease progresses. The most prevalent NPS in AD patients are apathy, depression, aggression, anxiety, and sleep disturbance^18–20^. Similar to anosognosia, the emergence of NPS in AD can exacerbate the condition and influence disease progression, prognosis, and caregiving needs, which in turn can have a significant impact on the quality of life for both patients with AD and their caregivers^21^.

Notably, due to the frequent co-occurrence between NPS and altered self-awareness, it has been suggested that they share a common neurobiological basis^22^. As the field moves toward identifying patients in earlier stages of AD during which there may be opportunities to preserve function, a better understanding of the relationship between anosognosia and NPS is critical for enabling patients to maintain their autonomy. Insight into the relationship between anosognosia and NPS may also contribute to interventions that could lessen the impact of each symptom on well-being.

Previous studies on this topic have found contradictory results. While some studies found no association between unawareness and NPS^23^, others have reported a significant correlation between unawareness and NPS; with the majority of previous studies investigating this relationship cross-sectionally^3^. The strongest relationship between anosognosia and NPS has been observed with apathy in cross-sectional studies^13,22,24–26^ and longitudinal studies^27^. Other studies have shown a relationship between anosognosia and agitation or irritability^7,28^ or psychosis^8,11,13,22^.

For some NPS, the direction of the association with anosognosia has provided mixed results. For example, some studies have shown that patients who demonstrate heightened awareness experience more depressive symptoms, possibly as an emotional and psychological reaction to being aware of their illness^7,13^, while other studies have found no association between the level of awareness and the presence of depressive symptoms e.g^29^.

To date, few studies have investigated the longitudinal association between awareness and NPS in patients with AD. In addition, previous studies were restricted to relatively small sample sizes or did not explore the association between anosognosia and distinct NPS. Moreover, the period of follow-up in most studies has been limited e.g^13^. For example, one study by Starkstein and colleagues, found that after a mean interval of 16 months, awareness was a predictor of the onset of depression and “emotional deficit” (including anxiety symptoms)^10^. The longitudinal relationships between anosognosia and other NPS are currently understudied.

To address this knowledge gap, the current study aimed to determine the (i) prevalence of NPS at baseline in dementia patients with and without anosognosia; and (ii) investigate the longitudinal association between anosognosia and onset of 12 different NPS.

## Methods

### Patients

The data used in our study was obtained from the Alzheimer’s Disease Neuroimaging Initiative (ADNI) database (https://adni.loni.usc.edu). ADNI is a global observational research project that began in 2003 as a longitudinal, multicenter study conducted at 59 sites across North America, enrolling cognitively normal, amnestic mild cognitive impairment (MCI), and AD dementia patients aged 55–94 years, and directed by the principal investigator Dr. Michael W. Weiner, MD. ADNI includes the expansion ADNI-GO (started in 2009) and extensions ADNI-2 (2011) and ADNI-3 (2016).

Study volunteers provide data using standardized protocols to ensure consistency. The collected data types include: Clinical data (demographics, physical exams, cognitive assessments, diagnostic categories); Genetic data (genome-wide association study, whole-genome sequencing); Neuroimaging data (MRI, PET scans); Biomarker data (cerebrospinal fluid, blood-based); Image analysis results (e.g. brain volumes, cortical thickness). Participants underwent PET scan using [18F] florbetapir (FBP) to determine amyloid beta (Aβ) burden in the brain. The FBP data is given as the standard uptake volume ratio (SUVr). The ADNI dataset provides global SUVr measurements (which account for the whole brain) for each individual. The global SUVr varied from 0.81 to 2.01, with a mean of 1.20 and a standard deviation of 0.24. Amyloid positivity or negativity was determined using a previously established threshold of 1.11 (i.e., those less than 1.11 were defined as amyloid negative)^30^.

The ADNI study uses a longitudinal design to collect data from patients at multiple time points, allowing for continuous monitoring instead of a one-time assessment. In the current study, patients were followed up to four years, with a total of five visits, including baseline assessments.

All ADNI patients provided written informed consent at the start of the study, and the project was authorized by each site’s institutional review board.

The general inclusion and exclusion criteria at the screening visit of ADNI were as follows: All patients had a modified Hachinski Ischemic Score of less than or equal to 4^31^; Patients were stable on allowed medications for at least four weeks prior to screening; a 15-item Geriatric Depression Scale score (GDS) below a score of 6; a study partner with 10 h per week of contact either in person or on the telephone and who could accompany the participant to the clinical visits; visual and auditory acuity adequate for neuropsychological testing; good general health with no diseases precluding enrollment; and at least 6 years of education or equivalent work history. The ADNI dataset includes clinical diagnoses that play a pivotal role in characterizing the cognitive status of patients in the study. These diagnoses are established by experienced clinicians, ensuring the accuracy and reliability of the information.

To be included in the current study, patients needed to undergo baseline assessments and at least one subsequent follow-up evaluation. Furthermore, all patients had to have amyloid PET, MMSE, NPI, and ECog variables available throughout the study.

### Exposure

#### Assessment of awareness (anosognosia versus non-anosognosia)

The Everyday Cognition (ECog) test was used to assess awareness^32^. The scale consists of 39 questions specifically developed to assess cognitive abilities in older adults. Questions are framed in the context of current performance compared to 10 years ago. The informant (study partner)-rated and self-rated versions of the ECog are composed of identical questions. Memory, Language, Visuo-Spatial, Attention, Planning, and Organization are the six cognitive domains measured by the ECog. Responses are on a Likert scale, with 1 indicating better or no change; 2, questionable or occasionally worse; 3, consistently a little worse; and 4, consistently much worse. The current stgudy focused on the memory sub-questionnaire (see supplementary Table 1), averaging the responses to the 8 questions. To classify patients with anosognosia, we used a modified approach to the one described in Starkstein and colleagues^27^, and as previously published in Wang and colleagues^33^. First, we calculated the average scores based on responses from both patients and study partners. Patients were classified as having anosognosia when their ECog average score for the study partner was ≥ 2.5 but the patient’s score was$\:\:\le\:\:$2.5. In contrast, patients were classified as not having anosognosia when both the study partner and the patient’s ECog score average was ≥ 2.5. Second, using longitudinal data, we only used patients who remained consistent across all longitudinal observations in either group. That is, the two groups (anosognosia / non-anosognosia) had to have a similar diagnosis at baseline and final visits, with no more than two consecutive diagnoses ‘deviating’ throughout the follow-up period.

### Outcomes

#### Assessment of neuropsychiatric symptoms

NPS presence was evaluated using the Neuropsychiatric Inventory (NPI), a questionnaire completed by study partners to assess the occurrence, severity, and frequency of specific NPS^34^.

The NPI covers twelve domains, including delusions, hallucinations, agitation/aggression, depression/dysphoria, anxiety, elation/euphoria, apathy/indifference, disinhibition, irritability/lability, aberrant motor behavior, sleep disturbances, and appetite and eating disorders. For each of these categories, study partners were initially asked if they observed the described symptom in the patient. If the symptom was observed, study partners were further questioned about its severity (rated as 1 = Mild, 2 = Moderate, or 3 = Severe) and frequency (rated as 1 = rarely, less than once per week; 2 = sometimes, about once per week; 3 = often, several times per week; and 4 = very often, once or more per day). If the symptom was not observed, the questionnaire moved on to the next category. The NPS assessment using the NPI was conducted at baseline and at subsequent follow-up visits for the ADNI patients.

The NPI total score was computed by summing the scores for each domain. These domain scores were calculated by multiplying the severity and frequency scores. The NPI total score can range from 0 to 144^34^. We analyzed follow-up data for up to 4 years. We used NPI total, as well as the presence or absence of each 12 NPS in our analysis.

### Statistical methods

Baseline observations of patients with and without stable anosognosia were compared. Continuous variables (age, education, MMSE and NPI total scores, and number of observations) were compared using the Wilcoxon rank sum test, while categorical variables (sex) were compared using Pearson’s Chi-squared test. The Cox proportional hazard ratio was employed to compare the hazard of developing each of the twelve distinct NPI items between patients with anosognosia and those without.

For each separate model (one for each NPS), patients who exhibited that specific NPS at baseline were excluded from the Cox models to be able to investigate the onset of symptoms. All Cox models were adjusted for age, sex, years of education, and MMSE. Given the small sample size, we are presenting our models without adjusting for multiple comparisons and thus acknowledge that they are exploratory. Statistical analyses were conducted using R version 4.2.0 (R Project for Statistical Computing).

## Results

### Baseline comparisons and correlations

**Fig. 1:**
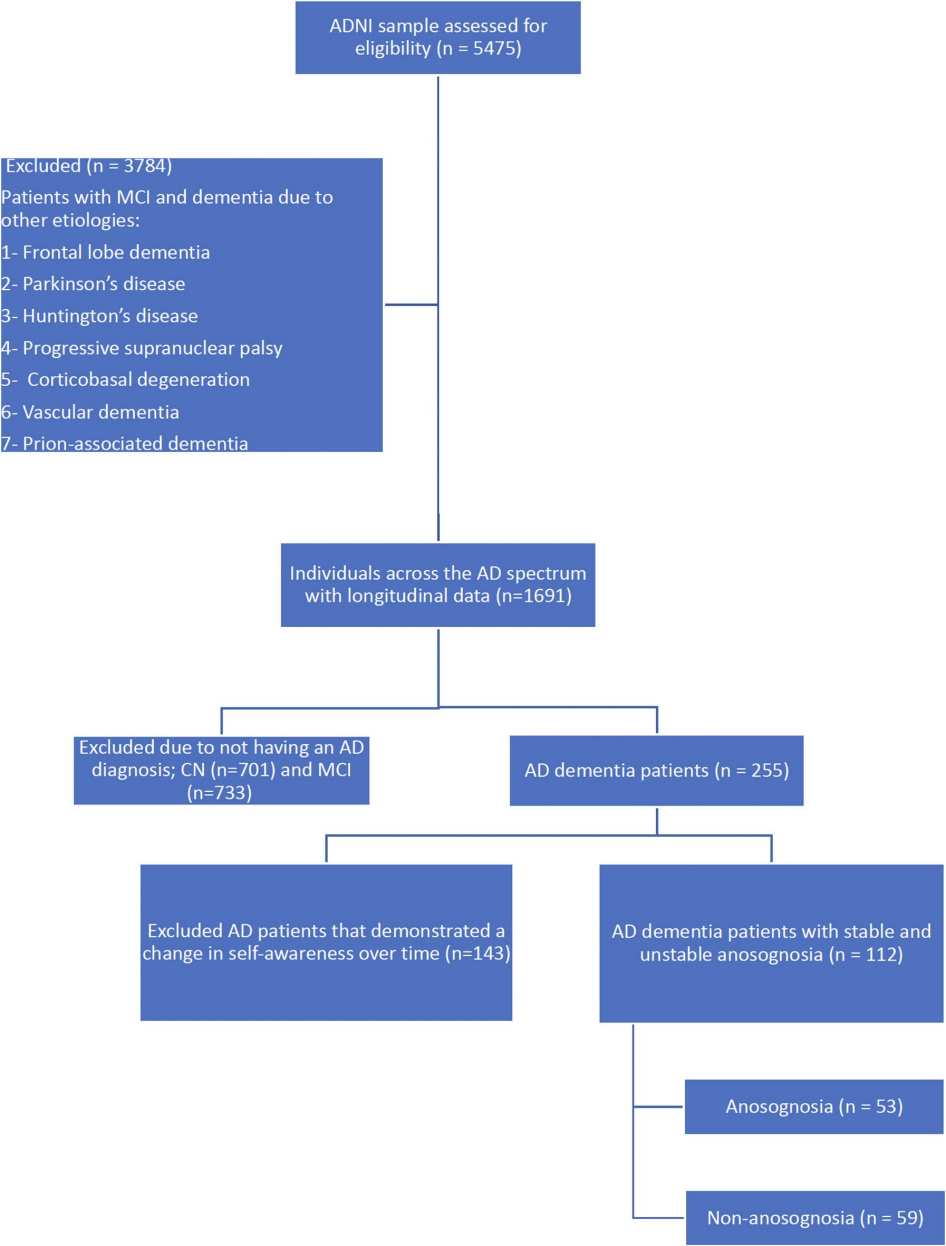
CONSORT diagram showing the selection flow of patients.

Overall, 112 patients met the eligibility criteria for this study (Fig. 1; Table 1). Of these, 47.3% of our sample exhibited anosognosia, i.e., 53 patients out of 112, and the remaining 52.7%, i.e., 59 patients, did not have anosognosia. The distribution of patients across the groups was comparable, indicating a balanced representation of the study.

**Table 1:** Baseline comparisons and correlations.

Characteristic	Overall, *N* = 112	Non-anosognosia, *N* = 59	Anosognosia, *N* = 53	*p*-value^1^
Number of visits, Median (IQR)	3 (2, 3)	3 (1)	3 (1)	0.7
Follow- up duration, Median (IQR)	1.03 (0.9, 1.9)	1.03 (0.9, 1.9)	1.02 (0.9, 1.1)	0.4
Age, years, Median (IQR)	76 (70, 80)	75 (68, 79)	77 (73, 81)	0.1
Sex (F), n (%)	46 (41%)	21 (36%)	25 (47%)	0.2
Education, years, Median (IQR)	16 (14, 18)	16 (14, 18)	16 (13, 18)	0.5
Amyloid +, n (%)	97 (87%)	51 (86%)	46 (87%)	0.9
MMSE^2^ score, Median (IQR)	23 (22, 25)	24 (22, 25)	23 (21, 26)	0.3
NPI^3^ Total, Median (IQR)	4 (1, 9)	3 (1, 7)	6 (1, 12)	0.1
Delusions., n (%)	9 (8%)	3 (5.1%)	6 (11%)	0.3
Hallucinations, n (%)	6 (5.4%)	4 (6.8%)	2 (3.8%)	0.7
Agitation, n (%)	41 (37%)	17 (29%)	24 (45%)	0.1
Depression, n (%)	40 (36%)	19 (32%)	21 (40%)	0.4
Anxiety, n (%)	25 (22%)	10 (17%)	15 (28%)	0.1
Euphoria, n (%)	0 (0%)	0 (0%)	0 (0%)	
Apathy, n (%)	37 (33%)	17 (29%)	20 (38%)	0.3
Disinhibition, n (%)	19 (17%)	7 (12%)	12 (23%)	0.1
Irritability, n (%)	38 (34%)	17 (29%)	21 (40%)	0.2
Motor, n (%)	16 (14%)	5 (8.5%)	11 (21%)	0.1
Sleep, n (%)	26 (23%)	11 (19%)	15 (28%)	0.2
Appetite, n (%)	28 (25%)	15 (25%)	13 (25%)	0.9

^1^Continuous variables (age, education, MMSE and NPI total scores, and number of observations) were compared using the Wilcoxon rank sum test, while categorical variables (sex) were compared using Pearson’s Chi-squared test.

^2^MMSE: Mini Mental State Examination.

^3^NPI: Neuro Psychiatric Inventory.

Across both groups, the median number of visits was 3, with an Interquartile Range (IQR) of 2–3 for both the overall and anosognosia groups and 2–4 for the awareness group. The difference in the median number of visits between groups was not statistically significant (*p*-value = 0.7). Similarly, there were no significant differences in the length of time patients were followed in each group (*p* = 0.4).

At baseline, we compared the two groups’ demographics and NPS (Table 1). The mean baseline age was 75 years (SD = 8) in the overall group, with a slightly higher mean age in the anosognosia group (76, SD = 8), though the difference was not statistically significant from the non-anosognosia group (*p* = 0.11). The percentage of females in the anosognosia group was 47%, but the difference was not statistically significant (*p* = 0.2) from the non-anosognosia group. The mean years of education were 16 years (SD = 3) overall and 15 (SD = 3) in the anosognosia group, with no significant differences between the groups (*p* = 0.5).

The distribution of Amyloid status (Amyloid- or Amyloid+) was not significantly different between the groups, and there were no significant differences in MMSE scores between the groups.

Furthermore, our investigation into NPS revealed that across the groups, the median NPI total scores displayed discernible differences, with patients without anosognosia having a median score of 3 (IQR: 1–7), while those with anosognosia had a higher median score of 6 (IQR: 1–12). However, the differences in NPI total scores between the two groups were only trending (*p* = 0.061).

Delving into specific NPI items, patients with anosognosia showed a trend toward higher frequency of agitation (45% vs. 29%) and motor symptoms (21% vs. 8.5%) at baseline. However, there were no trends or significant differences in the presence of other NPS between the groups.

### Survival analyses

In our Cox proportional hazard ratio models, we included 112 AD dementia patients, both with and without anosognosia. Cox regression models were performed for the twelve different NPI items (based on the presence or absence of symptoms), correcting for demographics, i.e., baseline age, sex, years of education, and baseline MMSE. For each model, we excluded patients with baseline symptoms of that specific NPS to investigate the rate of onset of NPS between the groups (Table 2).

**Table 2:** Proportion of patients exhibiting NPS at each study visit.

Symptoms	2nd visit *N* = 112	3rd visit *N* = 78	4th visit *N* = 26	5th visit *N* = 4
Delusions	103 (91%)	72 (92%)	25 (96%)	4 (100%)
Hallucinations	106 (94%)	74 (94%)	25 (96%)	3 (75%)
Agitation	71 (63%)	53 (67%)	19 (73%)	3 (75%)
Depression	72 (64%)	50 (64%)	18 (69%)	2 (50%)
Anxiety	87 (77%)	59 (75%)	18 (69%)	2 (50%)
Elation	112 (100%)	78 (100%)	26 (100%)	4 (100%)
Apathy	75 (66%)	53 (67%)	19 (73%)	1 (25%)
Disinhibition	93 (83%)	66 (84%)	22 (84%)	3 (75%)
Irritability	74 (66%)	50 (64%)	14 (53%)	3 (75%)
Motor	96 (85%)	67 (85%)	25 (96%)	4 (100%)
Sleep	86 (76%)	58 (74%)	18 (69%)	1 (25%)
Appetite	84 (75%)	58 (74%)	19 (37%)	

In Table 3, we present hazard ratios (HRs) and confidence intervals (CIs) for twelve NPS in the two groups, using both unadjusted and fully adjusted models, along with corresponding p-values. Apathy emerged as the sole symptom with a significant association in both the unadjusted (HR: 2.78, 95% CI: 1.37–5.62, *p* = 0.01) and fully adjusted (HR: 2.82, 95% CI: 1.32–6.05, *p* = 0.01) models. Of note, these models were exploratory. When we controlled for the false discovery rate using the BH method, no association was found between anosognosia and any of the outcomes of interest.

**Table 3:** Survival analysis of the neuropsychiatric symptoms.

	Neuropsychiatric symptoms	Models	HR^1^	95% CI^1^	*P*-value^1^	*P*-value (BH)	Global *P*-value^1^
1	Delusions	Unadjusted	1.41	0.51, 3.90	0.51	0.71	0.32
		Fully adjusted	1.58	0.56, 4.46	0.39	0.85	0.26
2	Hallucinations	Unadjusted	1.51	0.42, 5.47	0.53	0.71	0.81
		Fully adjusted	1.58	0.33, 7.42	0.57	0.85	0.11
3	Agitation	Unadjusted	0.68	0.28, 1.64	0.39	0.71	0.96
		Fully adjusted	0.77	0.28, 2.13	0.62	0.85	0.32
4	Depression	Unadjusted	1.12	0.44, 2.85	0.81	0.88	0.97
		Fully adjusted	1.23	0.45, 3.34	0.68	0.85	0.54
5	Anxiety	Unadjusted	0.77	0.36, 1.66	0.50	0.71	0.20
		Fully adjusted	0.85	0.38, 1.94	0.71	0.85	0.33
6	Elation	Unadjusted	0.30	0.03, 2.88	0.30	0.71	0.75
		Fully adjusted	0.02	0.00, 2.57	0.11	0.56	0.61
7	Apathy	Unadjusted	2.78	1.37, 5.62	0.01	0.54	0.45
		Fully adjusted	2.82	1.32, 6.05	0.01	0.09	0.56
8	Disinhibition	Unadjusted	0.54	0.22, 1.33	0.18	0.71	0.07
		Fully adjusted	0.46	0.16, 1.29	0.14	0.56	0.29
9	Irritability	Unadjusted	0.88	0.42, 1.87	0.75	0.88	0.75
		Fully adjusted	0.81	0.36, 1.83	0.61	0.85	0.76
10	Motor	Unadjusted	0.47	0.17, 1.27	0.14	0.71	0.33
		Fully adjusted	0.51	0.18, 1.44	0.20	0.61	0.62
11	Sleep	Unadjusted	1.11	0.31, 3.94	0.88	0.88	0.73
		Fully adjusted	0.91	0.23, 3.66	0.90	0.98	0.75
12	Appetite	Unadjusted	0.70	0.31, 1.59	0.39	0.71	0.73
		Fully adjusted	1.00	0.40, 2.53	0.99	0.99	0.15

^1^HR = Hazard Ratio, CI = Confidence Interval, Adjusted p-values presented in the table are corrected for age, sex education in years and MMSE, with false discovery rate controlled using the Benjamini-Hochberg (BH) method to address multiple comparisons. The global p-value assesses the overall proportional hazards assumption for the Cox proportional hazards model by correlating scaled Schoenfeld residuals for each covariate with time.

## Discussion

The objective of this research was to examine the association between self-awareness of memory decline and NPS in patients diagnosed with AD dementia. Based on previous findings, we hypothesized that unawareness of memory decline or anosognosia would correlate with a higher occurrence of NPS in AD dementia over time. Within our cohort, we found that 47.3% of patients had anosognosia, while 52.7% did not.

To classify patients with anosognosia, we used a modified approach to the one described in Starkstein et al.^27^. Using longitudinal data, we only used patients who remained consistent across all longitudinal observations in either group. In both groups (anosognosia/non-anosognosia), the classification had to be consistent between baseline and final observations, with no more than two consecutive ‘deviating’ observations during the follow-up period.

Delving into specific NPS at baseline, patients with anosognosia showed a trend toward higher rates of agitation (45% vs. 29%) and motor symptoms (21% vs. 8.5%).

Agitation in patients with AD has been linked to an elevated burden of neurofibrillary tangles (NFT). Specifically, NFT in the orbitofrontal cortex has been associated with agitation and specific aberrant motor behaviors, such as fidgeting, wandering, pacing, or rummaging^35^. Moreover, agitation in AD has been linked to atrophy in the frontolimbic regions, the right posterior cingulate, and the left hippocampus^36^.

The longitudinal trajectories of self-awareness are still a rather unexplored area. Some longitudinal studies have revealed that patients often rate their memory decline less severe than their study partners, both at baseline and over time, suggesting that the patient is experiencing anosognosia e.g^37^. However, a recent study by Clare and colleagues, found that with longer follow-up intervals and a discrepancy-based method for examining awareness, there is a stability in awareness over time despite an increase in dementia-related symptoms and progressing cognitive decline^38^.

A recent study examined the association between anosognosia and the frequency and development of NPS in patients with MCI and observed significantly earlier onset of seven of the 12 NPS in patients with anosognosia as compared to MCI patients without anosognosia^33^. These seven NPS included delusion, hallucination, agitation, apathy, disinhibition, irritability, and aberrant motor behavior^33^. In the current study, after excluding dementia patients with baseline NPS, and after adjusting for baseline age, sex, years of education, and MMSE as confounding factors, our findings from the exploratory Cox regression models revealed faster onset of apathy in patients with AD dementia and anosognosia compared to those without anosognosia. However, it should be noted that these results are exploratory and did not survive multiple comparison correction. Taken together these findings could indicate that the association between unawareness and NPS is more relevant in the prodromal stages of AD and less so in the dementia stages. Longer follow-up studies are needed to understand these complex interactions between change in self-awareness and the emergence of different NPS across the different AD stages.

While earlier research has established a cross-sectional association between anosognosia and symptoms like agitation e.g.^7^, our study builds upon this by examining the longitudinal dynamics of these associations. What distinguishes our research is its comprehensive investigation into the longitudinal dynamics between anosognosia and a wide range of NPS, including 12 items from the widely used NPI.

However, we acknowledge that this study has some limitations. First, our sample consisted primarily of highly educated participants. Second, the mean follow-up period of 1.5 years was limited and is not capturing the entire disease trajectory. Third, the relatively low baseline burden of NPS (as indicated by a low NPI total score of 7) may affect the generalizability of our results to patients with a higher symptom burden. Additionally, the stratification method in our exploratory study resulted in a smaller sample size, limiting the statistical power to detect associations and perform adjusted analyses, potentially leading to issues like residual confounding.

In our work, we gauged the prevalence of NPS through scores provided by the study partners. Simultaneously, we determined the presence of anosognosia by calculating the difference between the ECog questionnaire scores reported by the study partner and the patients themselves. We assumed that study partners could provide an objectively accurate assessment of the patients’ experiences. This assumption hinges on the study partners’ comprehensive understanding of the patients’ conditions, as well as the study partners’ own intact cognitive status. However, it is essential to recognize that study partner ratings might be influenced by various factors, including study partner/caregiver burden and emotional distress. Furthermore, patients may tend to underreport their symptoms due to fear or denial, and study partners may not always possess a complete understanding of the patients’ experiences. To address these potential sources of bias, future research endeavors might explore other measures of awareness. Using an alternative measure that assesses the difference between a patient’s subjective perception and objective evaluation could help mitigate biases and provide a more accurate reflection of the patient’s awareness status. Furthermore, in our study, we defined anosognosia by looking at discrepancy measurements over time. Although this resulted in a smaller sample size, we believe this approach made it possible to assess the onset of NPS in a more homogenous sample. However, we acknowledge that this approach does not provide details about the development of both symptoms over time. Given previous findings demonstrating that anosognosia develops in the predementia stages of AD^39–42^ we plan on looking at this question in a larger sample with patients over the whole spectrum of AD. In the current sample, 13% were amyloid-negative, suggesting other underlying causes of dementia. Given our small sample size for the current study, we were not able to run sensitivity analyses in the amyloid positive patients only. However, given that we did not observe any differences in amyloid burden between the patients with and without anosognosia, we do not expect that our results would have changed. However, future studies should look at the influence of amyloid or other AD pathologies on these relationships.

Another limitation of the study is the exclusion criteria applied in the ADNI study, which may have restricted the generalizability of the findings. These criteria excluded patients with clinically significant abnormalities in thyroid function tests, GDS scores higher than 6, unstable medical illnesses, contraindications for MRI or PET imaging, and residence in a skilled nursing facility. While these criteria aimed to ensure a similar group of patients in the study, they may have inadvertently excluded patients with certain comorbidities or conditions that could affect symptoms or treatment outcomes. However, upcoming phases of the ADNI study, such as ADNI-4, may loosen these exclusion criteria to improve the generalizability of the findings and allow the inclusion of patients with a broader range of comorbidities, enhancing the applicability of the results to real-world clinical settings^43^. Finally, psychotropic medication use may have influenced the onset or presence of NPS, and these data were not captured in detail in the ADNI dataset nor entered into our analyses, which presents a limitation. This information, if studied in a larger sample with longer prospective follow-up or in a clinical trial, could provide valuable insights into the potential confounding effects of medication on NPS development in AD dementia.

This study highlights the importance of tailored interventions and support strategies for patients with diminished self-awareness in AD, like psychoeducation, behavioral interventions, and pharmacological options, that can greatly benefit AD dementia patients and their caregivers. It emphasizes the need for targeted counseling, cognitive training, and rehabilitation, particularly for those with limited awareness, as these interventions have been shown to improve their well-being.

Long-term monitoring of awareness is crucial in AD, as regular assessments can detect shifts in awareness and anticipate the development of NPS. This early detection allows for timely intervention and management of these symptoms, improving the overall well-being of patients with AD.

Future research should adopt a more comprehensive approach to studying awareness in AD, integrating advanced neuroimaging techniques like functional magnetic resonance imaging (fMRI) and positron emission tomography (PET). These techniques may provide valuable insights into the underlying neural mechanisms associated with awareness, complementing the use of standardized awareness assessment methods. The extension of longitudinal observation periods can provide a more nuanced perspective on the dynamic interplay between awareness and NPS in AD.

## Conclusion

In conclusion, our research aimed to investigate the association between anosognosia and NPS in AD dementia patients. In this exploratory study, we observed a tendency toward a higher incidence of agitation and motor symptoms in those with anosognosia at baseline. In addition, our exploratory survival analysis revealed a significant difference for the onset of apathy between the groups, suggesting that patients with anosognosia have a faster onset of apathy as compared to patients without anosognosia. Overall, our study contributes to the understanding of the complex interplay between anosognosia and NPS in AD dementia. These findings highlight the potential relevance of anosognosia in clinical management and suggest that altered self-awareness may inform future intervention strategies. Further research is warranted to explain the underlying mechanisms driving these associations and to inform the development of targeted therapeutic strategies aimed at improving patient outcomes in this population.

## Supplementary Information

Below is the link to the electronic supplementary material.

Supplementary Material 1

## Data Availability

The datasets generated and/or analysed during the current study are available in the ADNI-LONI repository at the following link: https://adni.loni.usc.edu and thus, are available to the public to download.
