# Supplementary material for: Association between anosognosia and neuropsychiatric symptoms in Alzheimer’s disease dementia patients
Source: Sci Rep. 2025 Dec 4;16:316. doi: 10.1038/s41598-025-29569-z (PMC12769538; doi:10.1038/s41598-025-29569-z)
Supplement: Supplementary file 1 — Supplementary Material 1 [file 41598_2025_29569_MOESM1_ESM.docx]

**Supplementary Table 1**

| **ECog - Memory Domain** |  | | | |
| --- | --- | --- | --- | --- |
| **Questions** | Better or no change | Questionable or occasionally worse | Consistently a little worse | Consistently much worse. |
| Remembering a few shopping items without a list. |  |  |  |  |
| Remembering things that happened recently (such as outings, events in the news). |  |  |  |  |
| Recalling conversations a few days later. |  |  |  |  |
| Remembering where he/she has placed objects. |  |  |  |  |
| Repeating stories and/or questions. |  |  |  |  |
| Remembering the current date or day of the week. |  |  |  |  |
| Remembering he/she has already told someone something. |  |  |  |  |
| Remembering appointments, meetings, or engagements. |  |  |  |  |

Supplementary Table 1 shows The Everyday Cognition (ECog) test, Memory sub-questionnaire.
